# Metabonomic analysis of water extracts from Chinese and American ginsengs by ^1^H nuclear magnetic resonance: identification of chemical profile for quality control

**DOI:** 10.1186/1749-8546-7-25

**Published:** 2012-11-12

**Authors:** Pui Hei Chan, Ken YZ Zheng, Karl WK Tsim, Henry Lam

**Affiliations:** 1 Department of Chemical and Biomolecular Engineering, The Hong Kong University of Science and Technology, Clear Water Bay, Hong Kong, China; 2 Divison of Life Science and Centre for Chinese Medicine, The Hong Kong University of Science and Technology, Clear Water Bay, Hong Kong, China

## Abstract

**Background:**

With the gaining popularity of commercially prepared decoctions of herbal medicines on the market, an objective and efficient way to reveal the authenticity of such products is urgently needed. Previous attempts to use chromatographic or spectroscopic methods to identify ginseng samples made use of components derived from methanol extracts of the herb. It was not established that these herbs can be distinguished solely from consumable components, which are responsible for the clinical efficacy of the herb.

In this study, metabonomics, or metabolic profiling, based on the application of ^1^H-Nuclear Magnetic Resonance (NMR), is applied to distinguish the water extracts of three closely related ginseng species: *P. ginseng* (from two different cultivated regions in China), *P. notoginseng* and *P. quinquefolius*.

**Methods:**

A water extraction protocol that mimics how ginseng decoctions are made for consumption was used to prepare triplicate samples from each herb for analysis. High-resolution ^1^H NMR spectroscopy was used to acquire metabolic profiles of the four ginseng samples. The spectral data were subjected to multivariate and univariate analysis to identify metabolites that were able to distinguish different types of ginseng.

**Results:**

H NMR metabolic profiling was performed to distinguish the water extracts of *P. ginseng* cultivated in Hebei and Jilin of China, both of which were distinguished from extracts of *P. notoginseng* and *P. quinquefolius*, by unsupervised principle component analysis based on the entire ^1^H NMR spectral fingerprint Statistically significant differences were found for several discriminating features traced to common metabolites and the ginsenosides Rg1 and Rd, in the ^1^H NMR spectra.

**Conclusion:**

This study demonstrated that ^1^H NMR metabonomics can simultaneously distinguish different ginseng species and multiple samples of the same species that were cultivated in different regions. This technique is applicable to the authentication and quality control of ginseng products.

## Background

The *Panax* L. (Araliaceae) genus consists of 12 species, including 10 from Asia and two from North America. Three species are commonly used today: *Panax ginseng* C.A. Mey., known as Ginseng, *Renshen*, or Korean Ginseng; *Panax quinquefolius* L., known as American Ginseng or *Xiyangshen*; and *Panax notoginseng* (Burk.) F.H. Chen, known as Notoginseng or *Sanqi*. While these *Panax* species have different clinical efficacies in Chinese medicine, they share a great deal of similarity in their chemistry and gene sequences, making their authentication difficult.

Currently, quality control of these three ginsengs is based on the relative quantities of saponins [1]. In addition, the ginsengs are graded and priced according to their origins, ages, and morphological characteristics [1], which are mainly determined after visual or microscopic inspection by experts [2]. However, this morphological method is subjective, and cannot be applied to medicinal products in the form of slices, powders, or decoctions.

Genetically, the three *Panax* species closely resemble one another. For example, *P. ginseng*, *P. quinquefolius*, and *P. notoginseng* have similar DNA sequences [3] for nuclear ribosomal DNA, 5S rRNA spacer, and 18S rRNA. Random amplified polymorphic analysis can distinguish the *Panax* species [4,5], with restriction to the crude herbs instead of their extracts. Chemically, the important constituents of *Panax* roots, including ginsenosides Rb1, Rb2, Rd, Rg, and Re, are found in the roots of *P. ginseng*, *P. quinquefolius*, and *P. notoginseng*[6]. The presence of ginsenosides alone cannot offer conclusive evidence for species distinction. In 2006, Yang *et al*. [7] demonstrated the use of NMR metabonomics for quality control of commercial ginseng preparations. Similar metabonomic approaches were applied to distinguish the roots of *P. ginseng* and *P. quinquefolius* from different countries and ages by Kang *et al*. [8] and Lee *et al.*[1]. However, these previous attempts used methanol extraction, which is not typically used in ginseng preparation for consumption.

Therefore, this study aims to distinguish the *Panax* species roots from different cultivated regions in China and America in water extracts, that mimics the general way of ginseng consumption, to assess the potential of distinguishing ginseng decoctions by ^1^H NMR metabonomics.

## Methods

### Materials and reagents

All *P. ginseng* roots (Hebei-voucher# 10-5-23 and Jilin-voucher# 10-10-11) were obtained from their cultivated regions in China. *P. quinquefolius* roots (voucher# 10-8-12) were purchased from a local pharmacy and *P. notoginseng* roots (voucher# 10-9-26) were collected from Yunnan in China. The plant materials were collected in 2010, and authenticated by Dr. Tina T.X. Dong at Hong Kong University of Science & Technology according to their morphological characteristics [9]. The voucher specimens were deposited in the Centre for Chinese Medicine R&D at Hong Kong University of Science & Technology. All other reagents used in this study were of analytical grade (Sigma-Aldrich, USA).

### Sample preparation

Ginsengs were prepared using the extraction procedure optimized by Song *et al*. [10]. Briefly, each sample (1 g) was cut into granules, boiled in 8 volumes of water (w/v) for 2 h, and extracted twice. The extracts were dried by lyophilization and stored at −40°C. Five biological replicates of each of the four herbs were prepared and analyzed in the same manner.

### High-performance liquid chromatography (HPLC)

The water extracts of different ginsengs were first analyzed by HPLC fingerprinting, according to a previously described method [11], to confirm the effectiveness of water extraction.

### Sample preparation for NMR spectroscopy

Each extract (100 mg) was dissolved in 600 μL of sodium phosphate buffer (0.2 M Na_2_HPO_4_, 0.043 M NaH_2_PO_4_, 3 mM NaN_3_, 100% D_2_O, pH 7.4) with an internal standard, 0.1 mM sodium 3-(trimethylsilyl)propionate-2,2,3,3-d4 (TSP-d4). All particulate materials were removed by centrifugation (Prism, Labnet international, USA) at 13,000 x *g* for 1 min, and the supernatant was transferred to a standard 5-mm NMR tube. NMR spectra were acquired using a Bruker AV 400 MHz NMR spectrometer (Bruker Biospin, Rheinstetten, Germany) with a 5-mm PA BBO 400SB BBFO-H-D05 Z-gradient BB observe probe head, operating at 400.13 MHz ^1^H NMR frequency at 298 K. Gradient shimming was used to improve the magnetic field homogeneity prior to all acquisitions. ^1^H NMR spectra of the samples were acquired using a 1D NOESY pulse sequence (RD-90o-t1-90o-tm-90o-acquire) to generate a spectrum with a reduced residual solvent peak, 2-s relaxation delay, 100-ms mixing time, 20-ppm spectral width and 32000 acquired points. Each sample was run for 10 min. All spectra were Fourier-transformed, phase-corrected, and baseline-corrected manually.

### Statistical analysis

All data were integrated using the rNMR program [12] and normalized by TSP-d4 as an internal standard. The data were formatted in XML for import into Matlab version 2009b, (MathWorks, USA) and SIMCA-P version 12.0 (Umetrics, Sweden). Each ^1^H-NMR spectrum was *Pareto* scaled and divided into 1.3-K bins (bin width, 0.0084 ppm). The summed intensity in each bin was used as a data point for principal component analysis (PCA). Metabolites were identified using Chenomx Profiler, a module of Chenomx NMR Suite version 7.5, online databases (hmdb.ca [13] & bmrb.wisc.edu [14]) and a previous report [15]. Differences between groups were performed by the Student’s *t*-test and subsequent Bonferroni correction of *P* values.

## Results

The water extracts of different ginsengs were first analyzed by HPLC fingerprinting, according to a previously described method [11], to confirm the effectiveness of water extraction. The average ^1^H spectra of the ginsengs from different origins, including *P. quinquefolius* from Wisconsin in the United States, *P. ginseng* from Hebei and Jilin in China, and *P. notoginseng* from Yunnan in China, were obtained (Additional file 1: Figure S1). By visual inspection, the average ^1^H NMR spectra of the four herbs showed similar, but also different characteristics.

**Figure 1:**
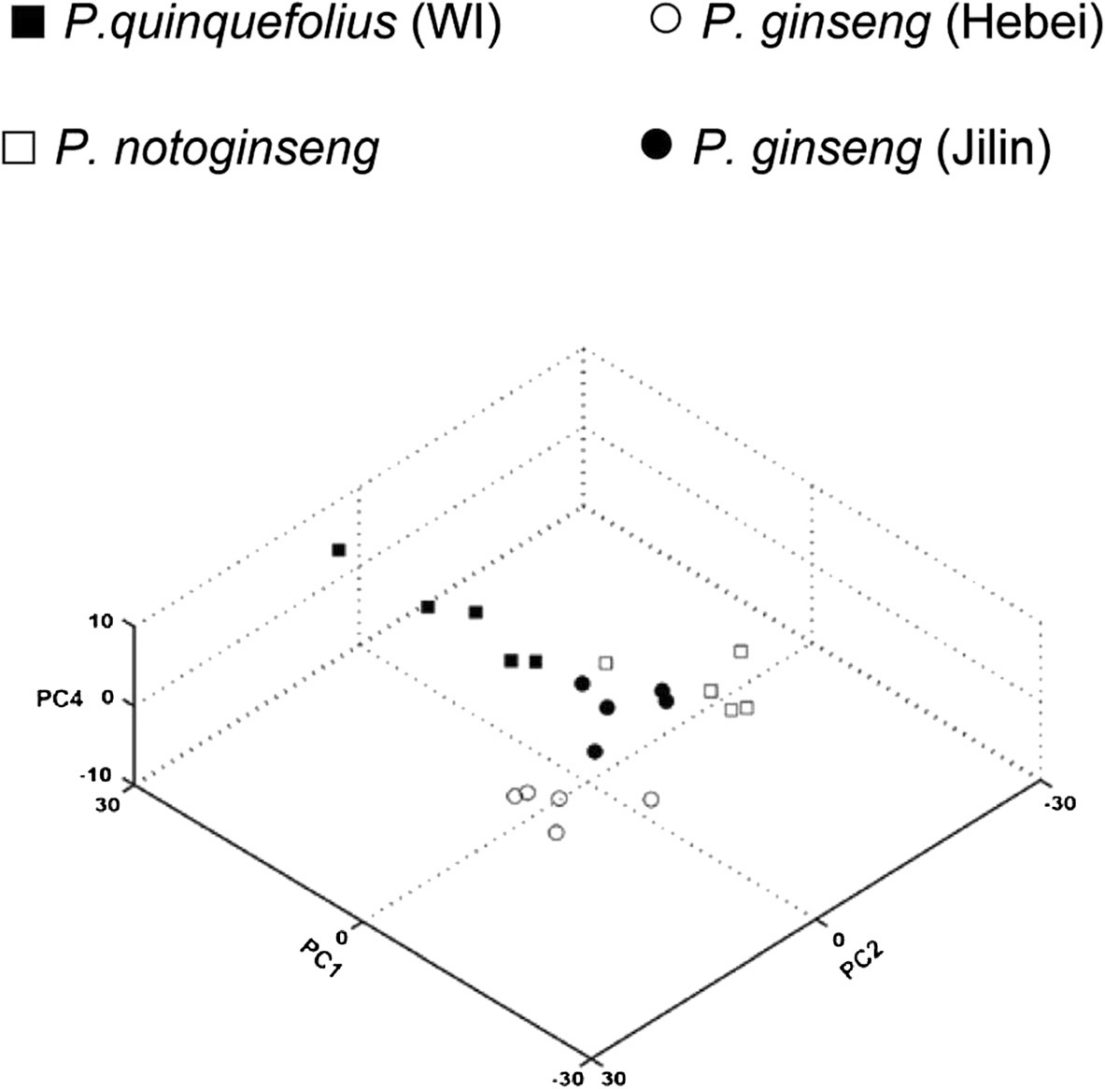
**Chemometric analysis by a 3D score plot of the PCA.** A score plot from pattern recognition (PCA) of the spectra is shown. The data sets are *Pareto* scaled. Overall, the plot can successfully distinguish the four groups of ginseng (N = 5). (PC1: R2 = 0.48, Q2 = 0.42; PC2: R2 = 0.71, Q2 = 0.59; PC4: R2 = 0.86, Q2 = 0.73).

The spectra were divided into data points and classified by PCA based on the sample origins. A PCA score plot, representing the relative position of each sample in the space of the principal components and maximizing the variance among all samples through linear combinations of sample features, revealed clusters of similar samples and individual samples with distinctive features (outliers). In this experiment, three principal components (PC1, PC2, and PC4) were required to conclusively classify all the spectra of the sample origins (Figure 1).

**Figure 2:**
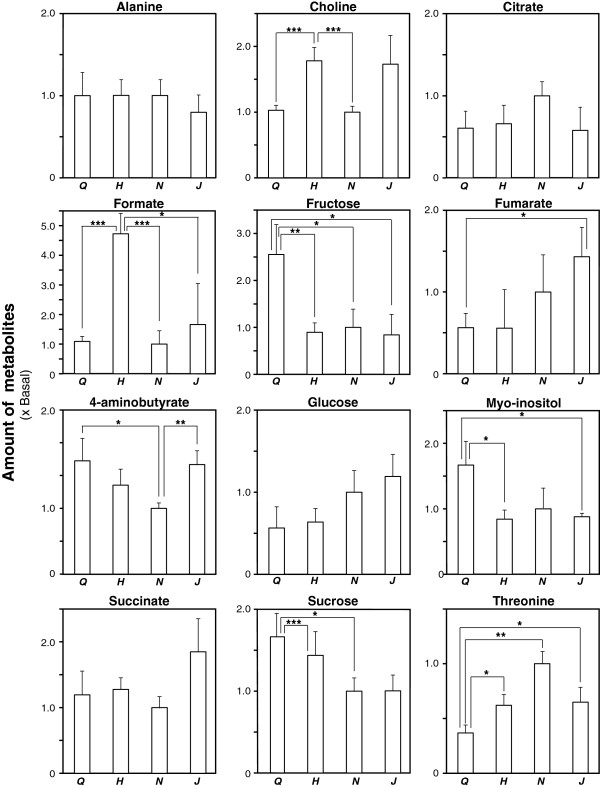
**Quantification of identified metabolites in the different ginseng extracts.** The peaks associated with identified metabolites in all of the ^1^H-NMR spectra were integrated to yield measures of the concentrations in the extracts relative to the mean value for *P. notoginseng* (basal level). The values are expressed as means ± SD (N = 5). *P ≤ 0.05, **P ≤ 0.01, ***P ≤ 0.001, by Student’s *t*-test (*P* values are Bonferroni-corrected for multiple testing.). Abbreviations: Q, *P. quinquefolius*; H, *P. ginseng* (Hebei); N, *P. notoginseng*; J, *P. ginseng* (Jilin).

Several regions of interest were isolated from the ^1^H-NMR spectra that distinguishd the herb origins, and were evaluated by Student’s *t*-tests on the differences in the metabolite concentrations between the herbs. A PCA loading plot (Additional file 2: Figure S2) showed that 3.4–4 ppm, a region associated with carbohydrates and sugars, had the most significant differences. The characteristic peaks associated with all metabolites were integrated (Figure 2, Additional file 3: Table S1), and showed that no metabolites could individually differentiate all ginsengs. Sucrose (5.42 ppm) was able to differentiate most of the ginsengs significantly, except for *P. quinquefolius* and *P. ginseng* (Hebei), and *P. notoginseng* and *P. ginseng* (Jilin).

**Figure 3:**
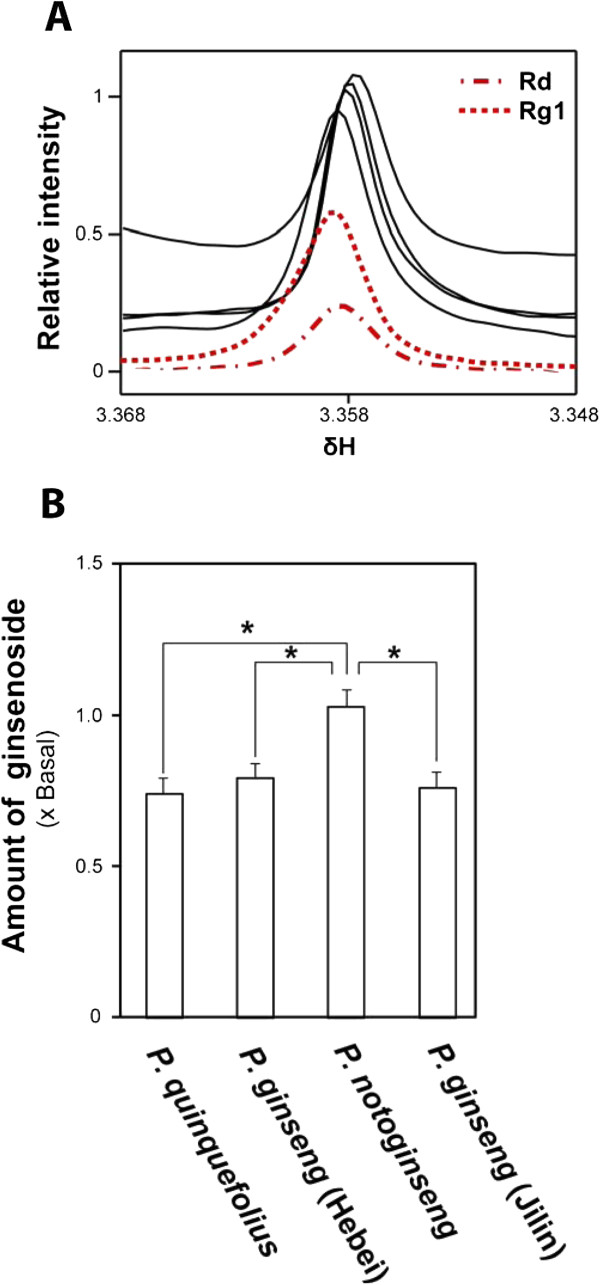
**Quantification of the ginsenoside peak at 3.358 ppm from different ginseng extracts.** (**A**) ^1^H NMR spectra of ginsenosides showing the resonance of Rg1 (solid) and Rd (broken line). (**B**) The ginsenoside peak at 3.358 ppm was integrated to yield the measurements of the ginsenoside concentrations in the extracts relative to the mean value of *P. notoginseng* (basal level). The values are expressed as means ± SD (N = 5). *P ≤ 0.05, **P ≤ 0.01, by Student’s *t*-test (*P* values are Bonferroni-corrected for multiple testing.).

Ginsenosides are the most important classes of compounds for the therapeutic effects of ginsengs, and are often used as chemical markers for quality control of ginsengs [6] The ^1^H NMR spectra of two major ginsenosides, Rg1 and Rd, were obtained (Additional file 4: Figure S3) and a well-resolved peak at 3.58 ppm, a location free from interfering signals from other identified metabolites in the ginseng water extract spectra, was identified (Figure 3A). All spectra exhibited a clearly resolved peak at 3.58 ppm. By assuming that the peak at 3.58 ppm arose from ginsenosides Rg1 or Rd and coincidentally from another unknown metabolite, we estimated that *P. notoginseng* contained a significantly higher amount of the ginsenoside than the other three ginsengs (Figure 3). However, the other three ginsengs were unable to be separated by the intensity of this peak, suggesting that the Rg1 and Rd concentration alone was insufficient to identify all the ginsengs.

## Discussion

This study differed from previous ginseng studies that used methanol as an extraction solvent [1,8]. The bioavailable chemicals of the four ginseng samples were directly observed by a water extraction protocol that resembles the commercial preparation of ginseng decoctions. Different extraction methods produce different quantities of metabolites. For example, comparing our metabolic profiles with those of Lee *et al*. [1], fewer metabolites were detected in the aromatic region (6–8 ppm). Our findings showed that *P*. *quinquefolius* had a significantly higher fructose concentration than *P*. ginseng, while Lee *et al.*[1] did not detect any significant difference. Our data also showed similar (in the case of Hubei-grown *P. ginseng*) or lower (in the case of Jilin-grown *P. ginseng*) concentrations in *P. quinquefolius* compared with *P*. *ginseng*, while Lee *et al.*[1] showed the opposite trend of much higher concentration of fumarate in *P. quinquefolius* than *P. ginseng*. Use of methanol extraction may not allow a direct demonstration of the chemical differences in the human-consumed components of these ginseng herbs.

The metabolite profiles from the ^1^H NMR spectra exhibited differences in the finer details for closely related ginseng species, and allowed measurements of different metabolites in an unbiased manner without prior chemical markers. The ^1^H NMR signal is directly proportional to the number of protons present, and the characteristic chemical shifts can identify and quantify many well-known metabolites, including amino acids, sugars, nucleotides, and other aromatic compounds [1]. In addition, the entire ^1^H-NMR spectrum can function as a fingerprint based on the content of metabolites from a biological sample, representing a valuable alternative to traditional methods in the absence of reliable chemical markers. PCA was used to identify differences in the ^1^H NMR spectra in an automated manner. When the group labels are unknown to the regression, this method is useful for outlier detection and detecting patterns and trends without prior knowledge. In this study, the four herbs were distinguished at once by the PCA, suggesting that this demonstration of the distinguishability may be more powerful than those in previous studies using supervised methods, such as Kang *et al*. [16] and Lee *et al.*[1].

No single metabolite was able to act as a biomarker for the classification of all four herbs. For example, fructose (4.21 ppm) was significantly different in *P*. *quinquefolius*, and can only be a potential biomarker for identifying *P*. *quinquefolius* but not the others, while sucrose (5.42 ppm) was able to differentiate most of the ginsengs significantly, but not *P. quinquefolius* and *P*. *ginseng* (Hebei), or *P. notoginseng* and *P. ginseng* (Jilin). These findings corroborate the findings from the PCA loading plot that the sugar region (3.4–4 ppm) is greatly discriminative. It is noted that choline was able to differentiate *P. quinquefolius* and *P*. *ginseng* (Hebei), and *P. notoginseng* and *P. ginseng* (Jilin). Taken together, choline and sucrose were able to distinguish the four ginsengs. A profiling approach can detect effective combinations of biomarkers automatically using ubiquitous metabolites.

To compare with the traditional method of discriminating samples based on known chemical markers, we attempted to identify peaks in the ^1^H NMR spectra that could correspond to two known ginsenosides. The ^1^H NMR spectra showed that *P. notoginseng* had a significantly higher amount of these ginsenosides than the other three herbs, consistent with a previous study [17]. In addition, the other three herbs were unable to be distinguished by this ginsenoside peak alone.

In this approach, the overall spectral fingerprint incorporating multiple markers, many of which have not previously been studied, was utilized successfully to distinguish the samples, in contrast to the traditional approach of relying on one or several known chemical markers. In contrast to previous work relying on different metabolite profiles obtained by methanol extraction, the water extraction used in the present study resembles that for commercial ginseng preparations.

## Conclusion

This study demonstrated that ^1^H NMR metabonomics can simultaneously distinguish different ginseng species and multiple samples of the same species cultivated in different regions. This technique is applicable to the authentication and quality control of ginseng decoctions.

## Abbreviations

PCA: Principle component analysis; TSP-d4: Sodium 3-(trimethylsilyl)propionate-2,2,3,3-d4.

## Competing interests

The authors declare that there are no competing interests

## Authors’ contributions

KWKT, HL and PHC conceived the study and designed the experiments. PHC and KZYZ conducted the experiments. PHC wrote the manuscript. All authors read and approved the final version of the manuscript.

## Supplementary Material

Additional file 1 **Figure S1.** Average ^1^H NMR spectra of the four ginseng extracts, P. quinquefolius, P. ginseng of Hebei, P. notoginseng, and P. ginseng of Jilin. The spectra represent the means of five replicates. By visual inspection, the average 1H-NMR spectra of the four herbs show a close resemblance, but also exhibit observable differences in the finer details. Click here for file

Additional file 2 **Figure S2.** PCA loading plot for the PC1 and PC2. The number next to the symbol shows the average chemical shift of the binned data. Click here for file

Additional file 3 **Table S1.** Statistical significance estimates (P values, Bonferroni-corrected) of differences of metabolite concentrations between pairs of samples by Student t-tests (N = 5). Click here for file

Additional file 4 **Figure S3.**^1^H NMR spectra of ginsenosides showing the resonance of Rg1 (black, solid) and Rd (red, broken line). Among the observed peaks, a well-resolved peak at 3.85 ppm, a location free from interfering signals in the ginseng extract spectra, is identified. Click here for file
