# Supplementary figures and images for: Metabonomic analysis of water extracts from Chinese and American ginsengs by 1H nuclear magnetic resonance: identification of chemical profile for quality control
Source: Chin Med. 2012 Nov 12;7:25. doi: 10.1186/1749-8546-7-25 (PMC3507782; doi:10.1186/1749-8546-7-25)

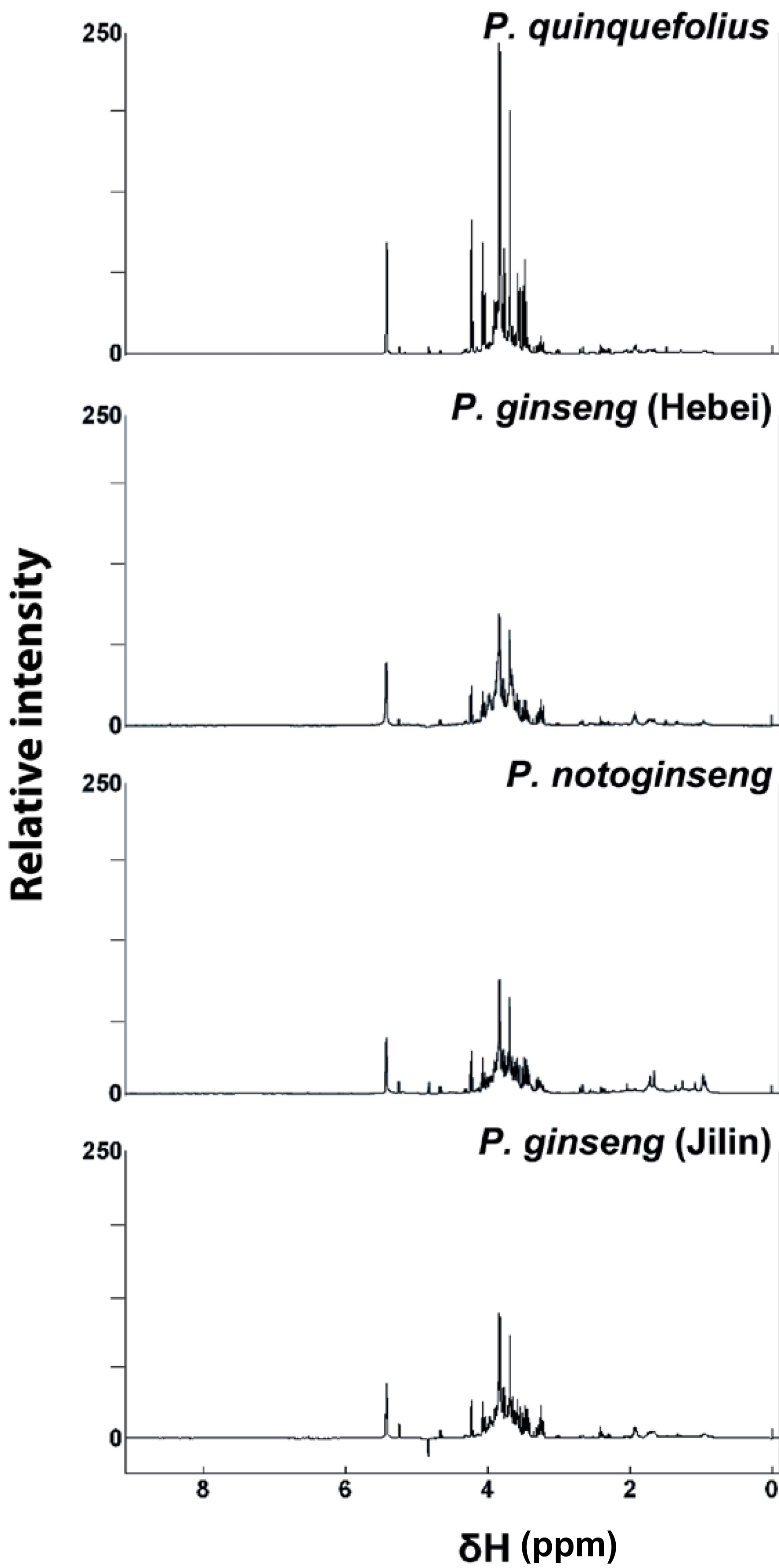

Supplement: Additional file 1 — Figure S1. Average 1H NMR spectra of the four ginseng extracts, P. quinquefolius, P. ginseng of Hebei, P. notoginseng, and P. ginseng of Jilin. The spectra represent the means of five replicates. By visual inspection, the average 1H-NMR spectra of the four herbs show a close resemblance, but also exhibit observable differences in the finer details. [file 1749-8546-7-25-S1.pdf]

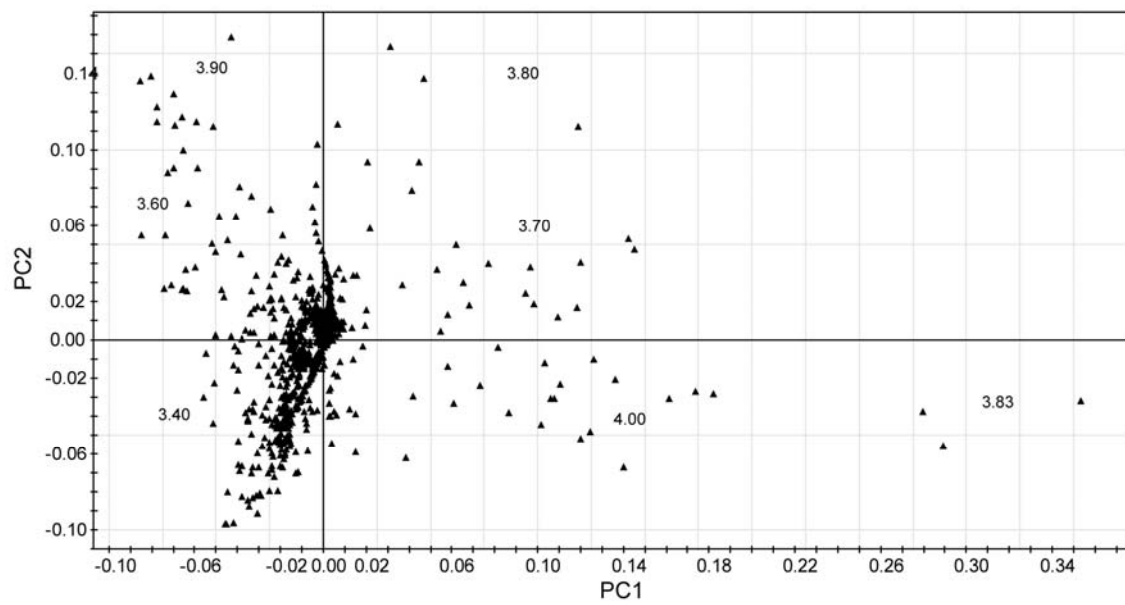

Supplement: Additional file 2 — Figure S2. PCA loading plot for the PC1 and PC2. The number next to the symbol shows the average chemical shift of the binned data. [file 1749-8546-7-25-S2.pdf]

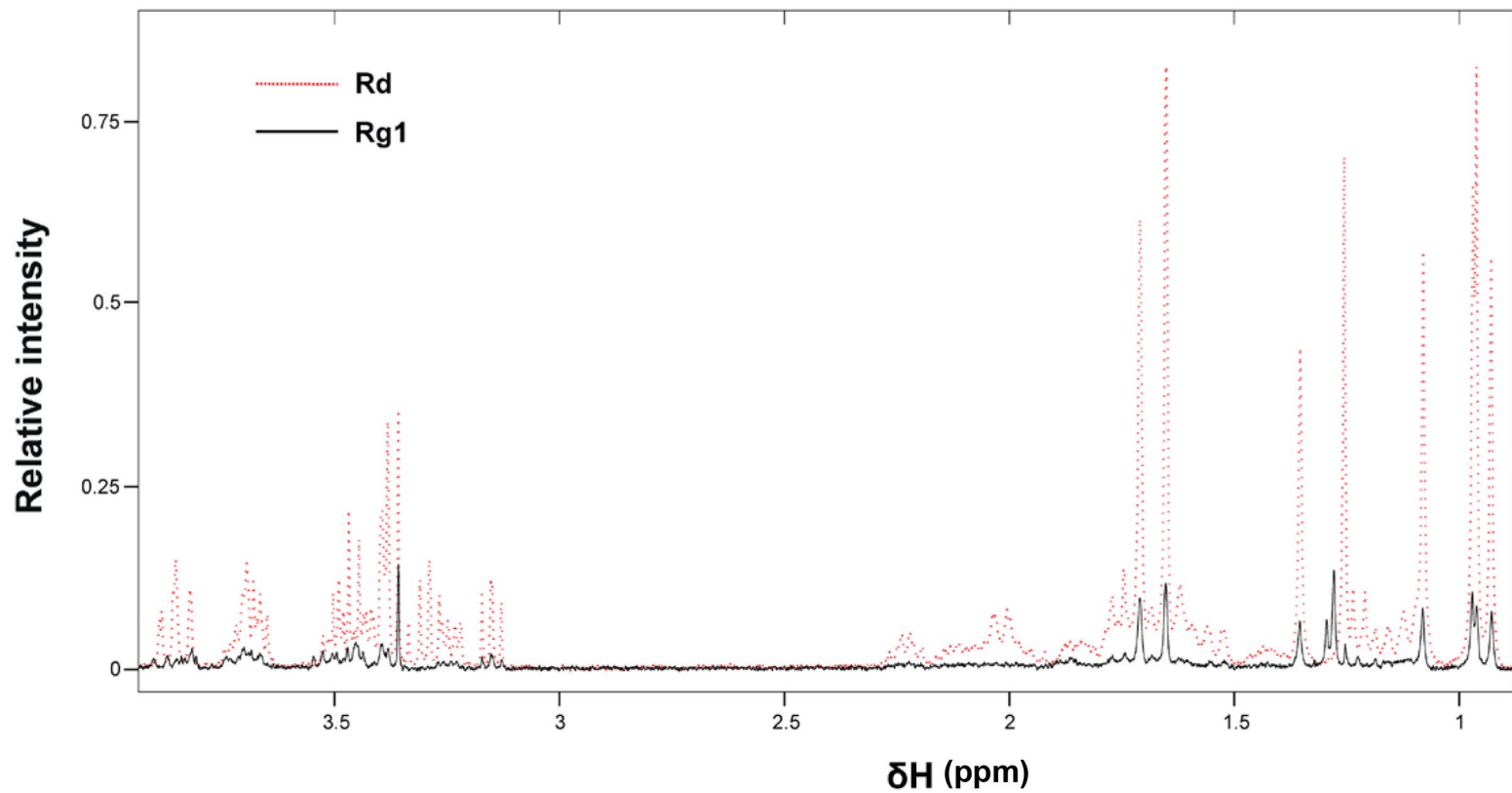

Supplement: Additional file 4 — Figure S3.1H NMR spectra of ginsenosides showing the resonance of Rg1 (black, solid) and Rd (red, broken line). Among the observed peaks, a well-resolved peak at 3.85 ppm, a location free from interfering signals in the ginseng extract spectra, is identified. [file 1749-8546-7-25-S4.pdf]
