# Supplementary material for: Metabonomic analysis of water extracts from Chinese and American ginsengs by 1H nuclear magnetic resonance: identification of chemical profile for quality control
Source: Chin Med. 2012 Nov 12;7:25. doi: 10.1186/1749-8546-7-25 (PMC3507782; doi:10.1186/1749-8546-7-25)
Supplement: Additional file 3 — Table S1. Statistical significance estimates (P values, Bonferroni-corrected) of differences of metabolite concentrations between pairs of samples by Student t-tests (N = 5). [file 1749-8546-7-25-S3.pdf]

Compounds	Q:H	Q:N	Q:J	H:N	H:J	N:J
**Alanine**	>1	>1	>1	>1	>1	>1
**Choline**	<0.001	>1	0.0783	<0.001	>1	0.0676
**Citrate**	>1	0.631	>1	0.964	>1	0.672
**Formate**	<0.001	>1	>1	<0.001	0.0313	>1
**Fructose**	0.0064	0.0298	0.0130	>1	>1	>1
**Fumarate**	>1	0.813	0.0154	>1	0.0980	>1
**4-aminobutyrate**	0.692	0.0230	>1	0.178	0.516	0.00309
**Glucose**	>1	0.333	0.0795	0.735	0.190	>1
**Ginsenoside**	>1	0.0198	>1	0.0480	>1	0.0158
**Myo-inositol**	0.0159	0.151	0.01497	>1	>1	>1
**Succinate**	>1	>1	0.405	>1	0.3858	0.218
**Sucrose**	<0.001	0.0295	0.0561	0.113	0.220	>1
**Threonine**	0.0204	0.00884	0.0363	0.150	>1	0.253
